# Evidence of Nitrogen and Phosphorus Limitation in Longleaf Pine Savanna Understories

**DOI:** 10.1002/ece3.71836

**Published:** 2025-09-21

**Authors:** Alyssa L. Young, Kathryn J. Bloodworth, Page A. Turner, Sally E. Koerner

**Affiliations:** ^1^ Department of Biology University of North Carolina at Greensboro Greensboro North Carolina USA; ^2^ Department of Entomology University of Maryland College Park Maryland USA; ^3^ North Carolina Wildlife Federation Raleigh North Carolina USA

## Abstract

Due to anthropogenic pressures, only 3% of the historic extent of the biodiverse longleaf pine ecosystem remains, much of which is degraded. Fire is necessary for maintaining longleaf pine savanna structure, function, and biodiversity; however, it also creates resource constraints, as nutrients are volatilized, especially in the already nutrient‐depleted soils of many longleaf pine savannas. Nutrient limitation and subsequent competition between plants can lead to changes in species diversity and productivity. Using a multiyear, chronic nutrient addition experiment, we explore how resource limitation influences restoration outcomes in longleaf pine savannas by affecting (1) productivity, (2) biodiversity metrics, and (3) community composition. In the field, we established a factorial N and P nutrient addition (10 g m^−1^ year^−1^) experiment. Nutrient additions were administered for 4 years, and plant composition and biomass were collected yearly. We measured biomass each year by functional group and calculated diversity metrics and community composition changes. Understory productivity typically increased with N and P additions, with N × P together having no additive effect. There were several significant interacting effects of nutrient addition treatments with year on our biodiversity metrics; however, the main nutrient addition effects were not significant for any biodiversity metric. Finally, community composition was significantly different in nutrient addition plots compared to control. Our results show that xeric Sandhill longleaf pine savannas exhibit distinct responses to fertilization, as fertilization led to increased productivity of the groundcover without reducing biodiversity. Low‐level, chronic nutrient inputs could influence understory structure in ways that help meet numerous management objectives, such as promoting fire spread or increasing forage availability. However, this may also present challenges, such as encouraging woody encroachment or threatening rare species. Our findings highlight the need for context‐specific approaches to longleaf pine savanna management and a careful evaluation of how nutrient dynamics interact with long‐term conservation and restoration goals.

## Graphical abstract

Due to anthropogenic pressures, only 3% of the historic extent of the biodiverse longleaf pine ecosystem remains, much of which is degraded. Fire is necessary for maintaining longleaf pine savanna structure, function, and biodiversity; however, it also creates resource constraints, as nutrients are volatilized, especially in the already nutrient‐depleted soils of many longleaf pine savannas. Nutrient limitation and subsequent competition between plants can lead to changes in species diversity and productivity. Using a multiyear, chronic nutrient addition experiment, we explore how resource limitation influences restoration outcomes in longleaf pine savannas by affecting (1) productivity, (2) biodiversity metrics, and (3) community composition. In the field, we established a factorial N and P nutrient addition (10 g m^−1^ year^−1^) experiment. Nutrient additions were administered for 4 years, and plant composition and biomass were collected yearly. We measured biomass each year by functional group and calculated diversity metrics and community composition changes. Understory productivity typically increased with N and P additions, with N × P together having no additive effect. There were several significant interacting effects of nutrient addition treatments with year on our biodiversity metrics; however, the main nutrient addition effects were not significant for any biodiversity metric. Finally, community composition was significantly different in nutrient addition plots compared to control. Our results show that xeric Sandhill longleaf pine savannas exhibit distinct responses to fertilization compared to other grasslands and savannas globally, as fertilization led to increased productivity of the groundcover without reducing biodiversity. Low‐level, chronic nutrient inputs could influence understory structure in ways that help meet numerous management objectives, such as promoting fire spread or increasing forage availability. However, this may also present challenges, such as encouraging woody encroachment or threatening rare species. Our findings highlight the need for context‐specific approaches to longleaf pine savanna management and a careful evaluation of how nutrient dynamics interact with long‐term conservation and restoration goals.

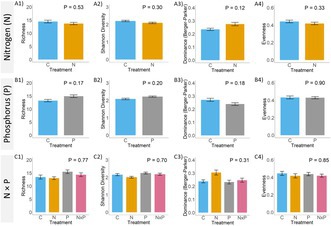

## 1 Introduction

Grassland and savanna ecosystems globally are regulated by factors such as climate, fire, and grazing, which all impact resource (e.g., water and nutrient) availability, with multiple factors often interacting to influence composition, structure, and function of ecosystems (Briggs et al. 2005; Strömberg 2011). For example, frequent fire limits woody encroachment, maintaining the open structure that typifies these ecosystems (Bond et al. 2005; Briggs et al. 2005; Bond and Keeley 2005), and invigorates groundcover biomass growth (Loudermilk et al. 2022). However, fire also creates nutrient constraints. When plant biomass is burned, elemental nutrients, such as nitrogen (N) and phosphorus (P) can be volatilized and lost from the system (Raison et al. 1985; Flannigan et al. 2009; Lavoie et al. 2010; Butnor et al. 2020). Fire can also alter soil chemical and physical properties more broadly, affecting nutrient cycling and availability over both the short and long term (Agbeshie et al. 2022). These changes are especially important in grassland and savanna ecosystems, where plant growth and primary productivity are frequently nutrient limited (Elser et al. 2007; Vitousek et al. 2010; Borer et al. 2017). Global grassland studies have shown that N and P addition is associated with significant decreases in richness and evenness, including the loss of legume species that are not primarily N limited as they can symbiotically fix their own nitrogen, and significant increases in productivity of most functional groups (not legumes) (Seabloom et al. 2015, 2021; Borer et al. 2017; Tognetti et al. 2021). Further, global studies have shown that as multiple, co‐limiting nutrients are added, the competitive trade‐off that contributes to species coexistence (Levin 1970) is eliminated, leading to often synergistic declines in diversity and increases in productivity, with the combined effect of multiple nutrients being more than the sum of each nutrient alone (Borer, Seabloom, et al. 2014; Fay et al. 2015; Simkin et al. 2016; Harpole et al. 2016; Borer et al. 2017; Midolo et al. 2019). While these studies assess changes in productivity and diversity, this does not capture the full picture of how plant communities can respond to fertilization. For example, there can be drastic changes in plant community composition (e.g., species gains/losses) with fertilization (Suding et al. 2005; Harpole and Tilman 2007; Clark and Tilman 2008), while changes in richness are not detected. Additionally, functional groups may respond differently to fertilization as resource requirements and acquisition strategies differ among growth forms. Therefore, slight changes in species abundance within functional groups could lead to major changes in ecosystem functioning (Avolio et al. 2014). This highlights the importance of studying the response to fertilization at the community level, considering changes within and among different functional groups.

Some savanna ecosystems are often left out of global studies because they are misclassified as forested ecosystems and exist in warmer, high precipitation climates, such as savannas of southeast Asia (Ratnam et al. 2016; Nguyen et al. 2019) and the southeastern United States (Pau et al. 2023). In comparison to these savannas, many well‐studied savannas experience similar precipitation (e.g., the mesic Australian and South American savannas) yet are cooler, or they occur within the same temperature range (e.g., drier African savannas), while receiving less precipitation (Pau et al. 2023). Despite the longleaf pine savanna ecosystem accounting for the entire grassy biome extent of the southeastern United States, historically spanning the majority of the North American Coastal Plain, longleaf pine savanna is often misclassified as a forested ecosystem due to the occasional high density of overstory trees (Frost 2006). While the overstory is dominated by the longleaf pine ( *Pinus palustris* ) tree, the understory is rich in various forb and graminoid species, making the longleaf pine savanna ecosystem among the most biodiverse ecosystems in the world (Walker and Peet 1984), although it has historically been overlooked as a biodiversity hotspot (Noss et al. 2015). Longleaf pine savannas are also distinct from other well‐studied grassland and savanna systems in that the sandy soils are extremely infertile (Clabo et al. 2020), there is minimal extant grazing pressure (Van Lear et al. 2005; Oswalt et al. 2012) compared to other grasslands, and the natural fire regime consisted of much more frequent lightning‐induced fires (every 1–3 years) (Frost 2006; Noss et al. 2015). Fire has been a critical factor governing longleaf pine savanna distribution, with reoccurring, low‐severity surface fires maintaining the structure, function, and understory biodiversity of longleaf pine savannas and preventing succession to hardwood forests (Waldrop et al. 1992; Mitchell et al. 2006). Frequent fire creates a cyclic process in longleaf pine savannas where the understory provides the fuel for fire spread. Fine fuels, such as dominant bunchgrasses ( *Aristida stricta* in the eastern part of the region and *Schizachyrium scoparium* in the western part), and forb species are primary sources of fuel in the understory, and fire in turn regulates vegetative responses in the understory and promotes production of fuels for the next fire (Mitchell et al. 2009; Fill et al. 2015, 2016). This understory vegetation‐fire feedback loop (Beckage et al. 2009, 2011; Fill et al. 2015) is a reinforcing loop that promotes the stability and persistence of longleaf pine savannas (Dixon et al. 2022).

Prior to European settlement, the longleaf pine ecosystem spanned ~92 million acres (Cerame et al. 2014). However, due to anthropogenic pressures (e.g., fire exclusion, conversion to row‐crop agriculture and pine plantations, urbanization), only ~5 million acres remain (Frost 2006; Harrington et al. 2013; Cerame et al. 2014; Kirschman et al. 2018), much of which is degraded (Oswalt et al. 2012). Further, the majority of longleaf pine savanna is found on private land, with 62% of *P. palustris* ‐dominated stands owned by nonindustrial private landowners (Oswalt et al. 2012). Private landowners often have diverse management goals (Butler and Leatherberry 2004) that include sources of revenue (e.g., timber and pinestraw production), as well as nonfinancial objectives (e.g., wildlife conservation, hunting, recreation, etc.). Therefore, restoration of ecosystem services within longleaf pine savanna understory in the context of multifunctional lands that hold both economic and conservation capacity is a priority (Oswalt et al. 2012; Dixon et al. 2022). Typically, longleaf pine savanna understory restoration has two interacting main targets—restoration of plant diversity and understory biomass that carries fire evenly across the landscape (Kirkman and Giencke 2017; Young and Koerner 2022). Likely, restoration for conservation will not be the only consideration for private landowners who may seek to increase productivity of the understory for multiple reasons such as beauty or wildlife habitat for hunting, in addition to fire spread. Management strategies therefore must differ when landowners have multifunctional goals, and research on understory restoration must account for differing management strategies that could include practices such as pinestraw raking, timber harvest, or fertilization.

To date, studies on resource limitation in longleaf pine savannas have found that P additions are not associated with changes in richness (at small spatial scales) (Brudvig et al. 2013), and while P addition increases legume species biomass (Tierney and Wurzburger 2024), its influence on other functional groups (e.g., graminoids) in longleaf pine understories has not been explored. The few studies on N additions in longleaf pine savannas have found decreases in richness, and either no impact on understory biomass of any functional group (Kirkman et al. 2016; Ament et al. 2018; Tierney and Wurzburger 2024), or increases in total biomass (Ford et al. 2008; Kirkman et al. 2016). While these few studies show independent effects of N and P, no studies to date have examined the relative importance of each, or how they may interact to impact understory biomass growth and diversity.

Using a multiyear, chronic nutrient addition experiment, we explore how N and P limitation influence longleaf pine savannas by affecting (1) understory productivity, (2) biodiversity metrics, and (3) community composition. We hypothesized that as N and P have both been found to limit grassland and savanna biomass, the singular addition of each nutrient would increase total, forb, graminoid, and woody biomass as the growth of all these groups is limited by both nutrients. We also hypothesized that legume biomass would not be affected by N addition alone but would increase with P addition as legumes can fix atmospheric N when in symbiosis with rhizobia and so are primarily only limited by P. We hypothesized that the interaction between N and P (N × P) would additively increase total, forb, graminoid, and woody biomass as both nutrients are limiting. Further, we hypothesized that the lack of an effect on legume biomass from N addition would be ameliorated by the addition of its limiting nutrient P, and legume biomass would be greatest with N × P addition. In terms of our biodiversity metrics, we hypothesized that richness, diversity, and evenness would decrease, and our metric of dominance would increase with all the nutrient addition treatments, as increases in biomass lead to competitive exclusion with fewer, more competitive species being favored. Finally, we hypothesized that plant community composition would be significantly changed under all nutrient addition treatments compared to control plots, as different species would be the best competitors under each nutrient addition treatment.

## 2 Methods

**FIGURE 1:**
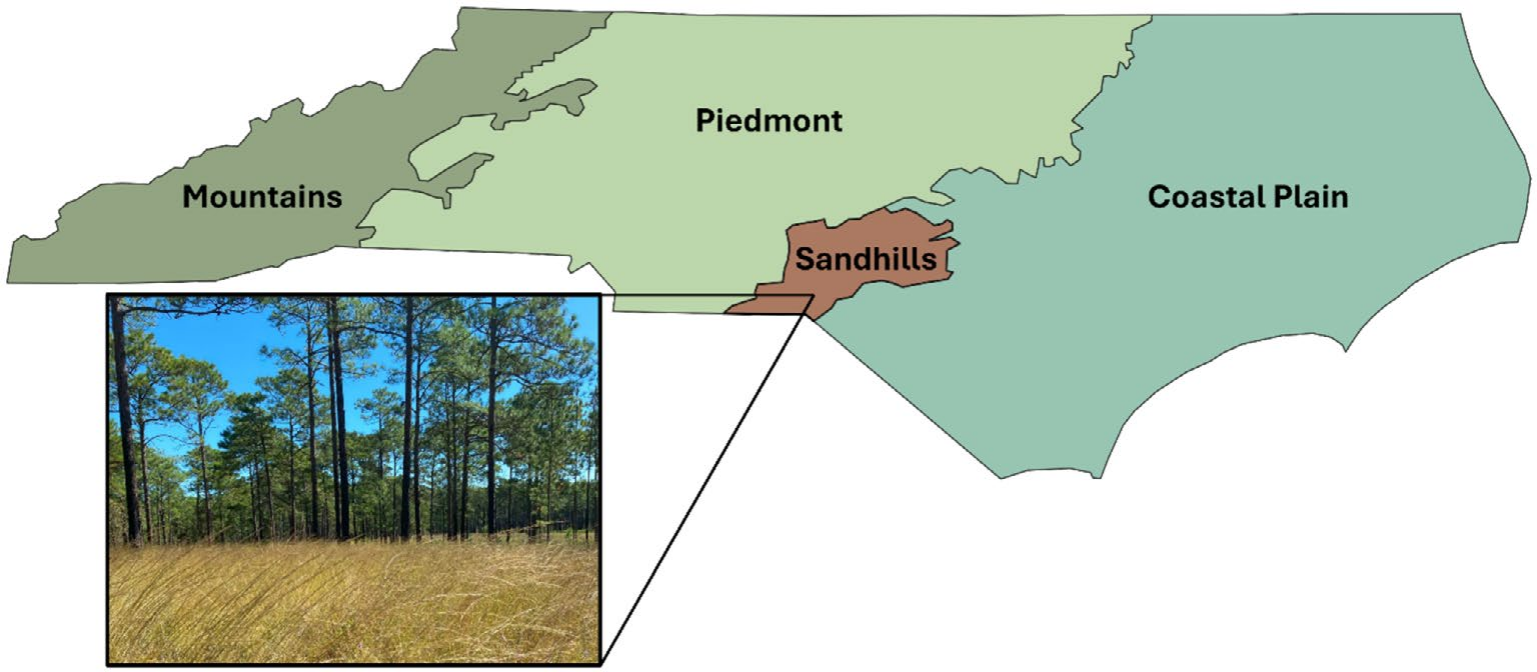
North Carolina map showing major ecoregions, with Sandhills shaded in brown. Insert depicts what the study site (and a typical longleaf pine stand) looks like in the Sandhill Gamelands.

This experiment was conducted in the Sandhills ecoregion of North Carolina (USA) from 2020 to 2023 on a portion of the 60,000‐acre Sandhills Gameland nature preserve (35.003, −79.567). The Sandhills Gameland consists predominantly of intact longleaf pine savanna, and our study site is characterized by dry, coarse, infertile sands (sandy Ultisols), a longleaf pine tree overstory (38 cm average diameter at breast height—*Young unpublished data*), and an understory of grasses, forbs, and scrub oaks (e.g., *Quercus laevis* , *Q. marilandica* , *Q. margarettae* ) (Figure 1). The site has a history of reoccurring, low intensity prescribed fires every 2–3 years in spring or early summer, with one burn occurring in early spring in the pretreatment year (2019) and again in early spring of treatment year 2 (2021). Mean annual precipitation during the 4 years of the experiment was 1197 mm and mean annual temperature was 18.3°C, ranging from 12.3°C to 25.3°C (NOAA 2024). At this field site (1050 m^2^ footprint), we followed the Nutrient Network protocols for site establishment and data collection (Borer, Harpole, et al. 2014). We established three blocks within the study site, each of which consisted of four, randomly assigned, 25 m^2^ plots that received nothing, or nutrient addition treatments of either commercial N (urea: 44‐0‐0), P (triple super phosphate: 0‐46‐0), or N × P, with each nutrient applied at a rate of 10 g m^−1^ year^−1^. For each nutrient addition, the 25 m^2^ plot was visually split into quarters, with each quarter receiving 25% of the total required nutrients by weight to enable an even spread of the granular nutrients across the entire plot. Nutrient additions were administered yearly for 4 years during the early growing season. While there was an additional drought treatment, drought had no ecologically significant effects, so all further mention has been confined to the supplemental material (Appendix S1, Appendix S2: Table S1, Appendix S3: Table S2). Plant community composition was sampled in the early and late growing season within a permanently marked 1 m^2^ area within each plot close to where aboveground biomass was sampled. Composition was estimated visually as absolute percent areal cover of each plant (Daubenmire 1959), so that total cover was often over 100%, providing a realistic depiction of complex and layered longleaf pine savanna understories. For each species, the maximum cover across seasonal sampling points was used in analyses. Each year during the late growing season, aboveground biomass was clipped in two, 0.1 × 1 m strips, sorted to functional group (i.e., forb, graminoid, legume, woody), dried at 60°C for at least 48 h, weighed in grams, and scaled up to represent 1 m^2^.

We used linear mixed models (package: *lme4*; Bates et al. 2015) with “block” and “plot” included as random effects, to test the main and interacting effects of N, P, year, and their interactions on graminoid biomass, non‐leguminous forb (hereafter called “forb”) biomass, legume biomass, non‐leguminous woody (hereafter called “woody”) biomass, total biomass (the sum of forb, graminoid, legume, and woody biomass), plant species richness, Shannon diversity index, Berger–Parker dominance index, and evenness (Evar) (Smith and Wilson 1996). We included year as a factor in our models because we expect that community‐level responses to fertilization would magnify through time, and to account for any variation due to year to year fluctuations in abiotic conditions at our site. To highlight the sole response of each dependent variable to our treatments, we do not show significant interactions with year in our figures. For each model, when there was a significant interaction among main effects, post hoc pairwise comparisons were calculated with Benjamani–Hochberg adjustments using the package *emmeans* (Lenth et al. 2024). For each model, we used the *DHARMa* package (Hartig F., 2024) to calculate residuals using the “residuals” function, conduct a non‐parametric dispersion test using the “testDispersion” function, and test for residual outliers using the tests within the “plot.DHARMa” function. We further assessed normality of the residuals using the “ols_test_normality” function for Shapiro–Wilk, Kolmogorov–Smirnov, Anderson–Darling, and Cramer–von Mises tests from the *Olsrr* package (Hebbali 2020). Berger–Parker dominance, graminoid biomass, forb biomass, woody biomass, and total biomass were all square root transformed, and legume biomass was transformed by taking the natural log and adding one (log1p) to achieve approximate normality and homoscedasticity. To assess compositional differences among nutrient addition treatments, we calculated Bray–Curtis dissimilarity for each pairwise combination of treatment plots using the “metaMDS” function and conducted a permutational multivariate analysis of variance (PERMANOVA) test on that distance matrix using the “adonis2” function from the *vegan* package (Oksanen et al. 2022) with treatments and year as fixed effects and block representing strata. A permutational test for homogeneity of variance (permDISP) was then performed to test for differences in plot replicate community similarity within nutrient addition treatment groups. We then used the “multipat” function from the *indicspecies* package (De Cáceres and Legendre 2009) to identify indicator species significantly associated with each treatment group. Finally, to track changes in communities in terms of the abundance of functional groups, we calculated the relative abundance of species in each year and for each plot. Those values were summed within functional groups and averaged across blocks. Then, functional groups were ranked and displayed as rank abundance curves (Avolio et al. 2019) with the first rank being the most abundant functional group within treatments across blocks, and fourth being least abundant. All calculations and analyses were conducted in the statistical program R version 4.2.3 (R Core Team 2023) (*α* = 0.05, with results 0.05 < *p* < 0.1 reported as marginally significant).

## 3 Results

### 3.1 Productivity

**FIGURE 2:**
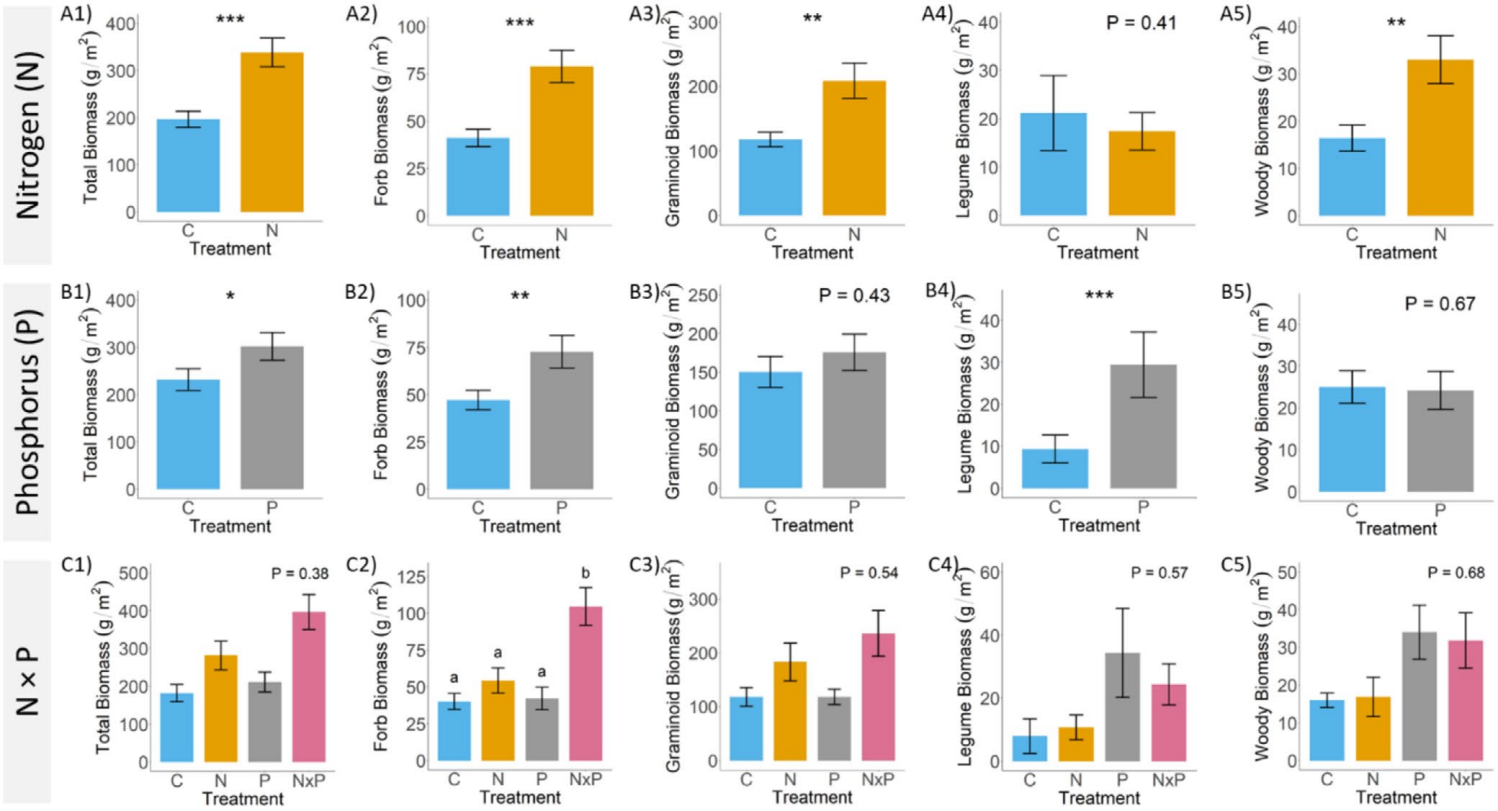
Mean and standard error of total (sum of forb, graminoid, legume, and woody biomass), forb, graminoid, legume, and woody biomass in response to N (A1–A5), P (B1–B5), and N × P (C1–C5) additions. Significant differences between control and N or P treatments are denoted with * and represent *p* < 0.10, *p* < 0.05, and *p* < 0.001 for one, two, and three asterisks, respectively. Significant differences between control, N, P, and N × P (panels C1–C5) are represented by letters when relevant. *p*‐values are provided for each panel where there were nonsignificant effects of treatments. Colors represent the different treatments (control = blue, nitrogen (N) addition = gold, phosphorus (P) addition = gray, and N × P addition = pink).

Compared to control, N addition was associated with a significant increase in total (53% greater, *p* = 0.002), forb (63% greater, *p* = 0.002), graminoid (56% greater, *p* = 0.02), and woody (67% greater, *p* = 0.01) biomass (Table 1, Figure 2A1–A3,A5, respectively), and had no significant effect on legume biomass (*p* = 0.41; Table 1, Figure 2A4). There was a significant interaction of N and year for forb biomass (*p* = 0.04, Table 2), with N addition associated with a 44% increase in year 2, a 39% increase in year 3, and a 54% increase in year 4 compared to control. P addition was associated with a marginally significant greater total biomass (26% greater, *p* = 0.09), a significantly greater forb (43% greater, *p* = 0.04) and legume (104% greater, *p* = 0.008) biomass (Table 1, Figure 2B1,B2,B4, respectively), and had no significant effect on graminoid (*p* = 0.43) or woody (*p* = 0.67) biomass (Table 1, Figure 2B3,B5, respectively). Finally, the interaction between N and P (N × P treatment) was associated with significantly increased forb biomass (*p* = 0.03), with N × P biomass being 89% greater than control, 63% greater than with N addition alone, and 85% greater than with P addition alone (Table 1, Figure 2C2); the combined addition of N × P had no significant effect on total (*p* = 0.38), legume (*p* = 0.57), or woody (*p* = 0.68) biomass (Table 1, Figure 2C1,C4,C5, respectively). Finally, there was a significant interaction between N × P and year for graminoid biomass (Table 2, *p* = 0.05), with graminoid biomass in year 2 being 62% greater with N × P addition compared to P addition (*p* = 0.01); apart from the interaction with year, there was not a significant interaction between N and P for graminoid biomass (*p* = 0.54, Table 2, Figure 2C3).

**TABLE 1:** Main and interacting effects of N, P, and year (fixed effects) from linear mixed models on total biomass (sum of forb, graminoid, legume, and woody biomass), forb biomass, graminoid biomass, legume biomass, and woody biomass.

Effect	Total biomass (*r* ^2^ *C* = 0.49)			Forb biomass (*r* ^2^ *C* = 0.45)			Graminoid biomass (*r* ^2^ *C* = 0.46)			Legume biomass (*r* ^2^ *C* = 0.49)			Woody biomass (*r* ^2^ *C* = 0.48)		
	df	*F*	*p*	df	*F*	*p*	df	*F*	*p*	df	*F*	*p*	df	*F*	*p*
Year	3, 58.4	2.80	**0.05**	3, 58.9	0.76	0.52	3, 57.7	4.32	**0.01**	3, 59.3	7.90	**< 0.001**	3, 59.0	10.69	**< 0.001**
N	1, 17.2	13.94	**0.002**	1, 17.8	13.30	**0.002**	1, 16.6	6.34	**0.02**	1, 20.1	0.71	0.41	1, 19.8	7.40	**0.01**
P	1, 17.2	3.29	**0.09**	1, 17.8	5.02	**0.04**	1, 16.6	0.64	0.43	1, 20.1	8.54	**0.008**	1, 19.8	0.19	0.67
Year × N	3, 58.4	1.52	0.22	3, 58.9	2.99	**0.04**	3, 57.7	0.57	0.64	3, 59.3	0.49	0.69	3, 59.0	1.08	0.37
Year × P	3, 58.4	0.88	0.46	3, 58.9	0.63	0.60	3, 57.7	1.22	0.31	3, 59.3	0.34	0.80	3, 59.0	0.34	0.80
N × P	1, 17.2	0.81	0.38	1, 17.8	5.39	**0.03**	1, 16.6	0.40	0.54	1, 20.1	0.34	0.57	1, 19.8	0.17	0.68
Year × N × P	3, 58.4	1.84	0.15	3, 58.9	1.25	0.30	3, 57.7	2.77	**0.05**	3, 59.3	0.16	0.92	3, 59.0	1.47	0.23

*Note:* Conditional *r* ^2^ (*r* ^2^ *C*) is included in the header for each model, and block and plot were included as random effects. Significant (*p* < 0.05) and marginally significant *p*‐values (*p* < 0.10) are bolded.

**TABLE 2:** Main and interacting effects of N, P, and year (fixed effects) from linear mixed models on species richness, Shannon diversity index, Berger–Parker dominance index, and evenness (Evar).

Effect	Richness (*r* ^2^ *C* = 0.82)			Shannon diversity (*r* ^2^ *C* = 0.81)			Berger–Parker dominance (*r* ^2^ *C* = 0.59)			Evenness (*r* ^2^ *C* = 0.59)		
	df	*F*	*p*	df	*F*	*p*	df	*F*	*p*	df	*F*	*p*
Year	3, 60	6.44	**< 0.001**	3, 60	5.89	**0.001**	3, 60	1.49	0.23	3, 60	2.69	**0.05**
N	1, 18	0.42	0.53	1, 18	1.16	0.30	1, 18	2.70	0.12	1, 18	1.01	0.33
P	1, 18	2.00	0.17	1, 18	1.76	0.20	1, 18	1.94	0.18	1, 18	0.02	0.90
Year × N	3, 60	2.10	0.11	3, 60	4.39	**0.01**	3, 60	2.88	**0.04**	3, 60	0.65	0.58
Year × P	3, 60	2.00	0.12	3, 60	1.07	0.37	3, 60	1.01	0.39	3, 60	1.13	0.34
N × P	1, 18	0.09	0.77	1, 18	0.15	0.70	1, 18	1.11	0.31	1, 18	0.04	0.85
Year × N × P	3, 60	1.43	0.24	3, 60	0.02	1.00	3, 60	0.37	0.78	3, 60	3.41	**0.02**

*Note:* Conditional *r* ^2^ (*r* ^2^ *C*) is included in the header for each model, and block and plot were included as random effects. Significant (*p* < 0.05) and marginally significant *p*‐values (*p* < 0.10) are bolded.

### 3.2 Biodiversity Metrics

**FIGURE 3:**
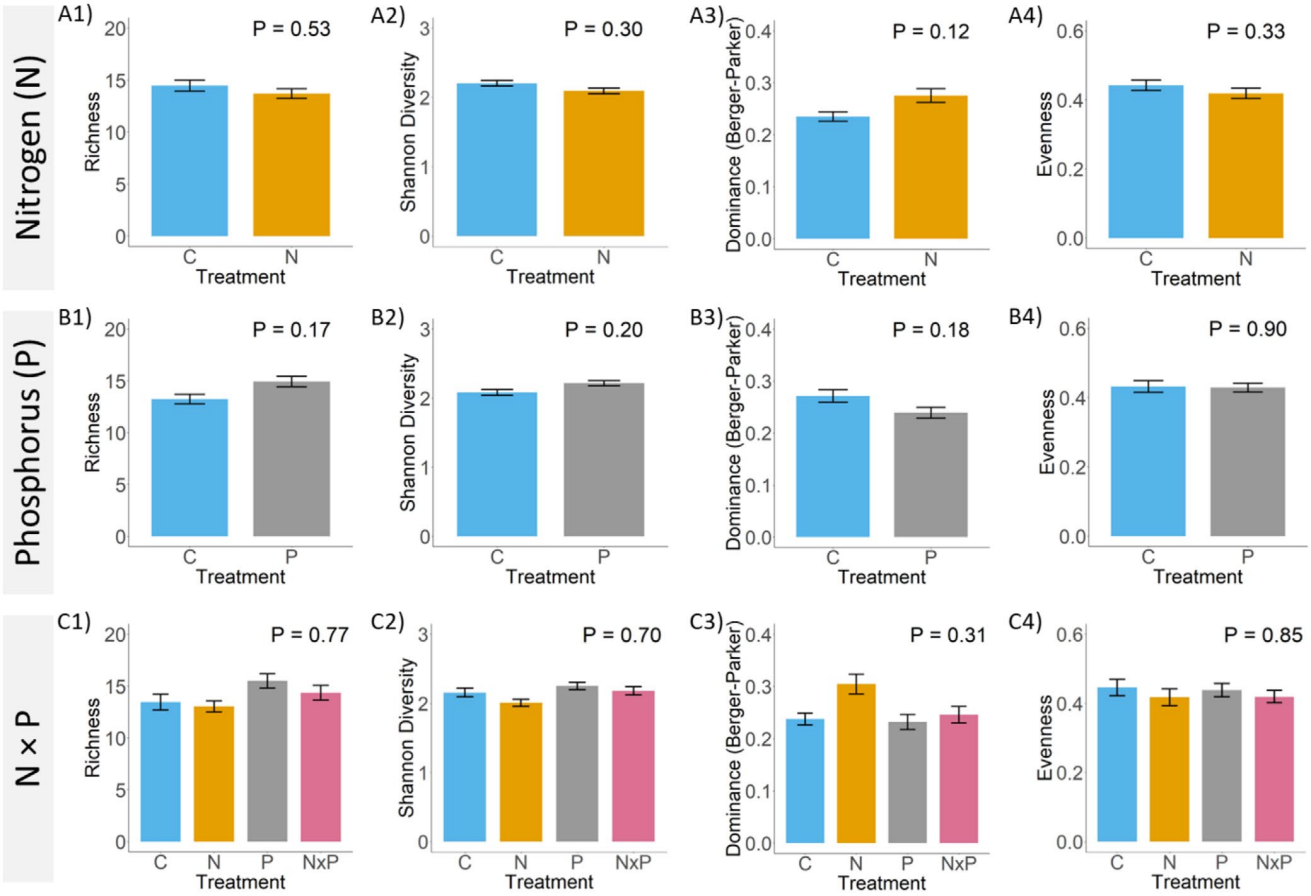
Mean and standard error of richness, Shannon diversity index, Berger–Parker dominance index, and evenness (Evar) in response to N (A1–A4), P (B1–B4), and N × P (C1–C4) additions. *p*‐values are included for each panel. Colors represent the different treatments (control = blue, nitrogen (N) addition = gold, phosphorus (P) addition = gray, and N × P addition = pink).

There were no significant main or interacting effects of N or P on any of our biodiversity metrics (richness, Shannon diversity, Berger–Parker dominance, and evenness) (Table 2, Figure 3 all panels); however, there were several interactions of our main nutrient addition treatments with year (Table 2). Shannon diversity was marginally significantly lower in years 3 (9% lower, *p* = 0.08) and 4 (10% lower, *p* = 0.08) with N addition, and Berger–Parker dominance was significantly greater in year 3 (32% greater, *p* = 0.01) and marginally significantly greater in year 4 (22% greater, *p* = 0.07) with N additions. Finally, for evenness, there was a significant interaction between N × P and year for evenness (Table 2, *p* = 0.02), but upon exploration, there were no significant post hoc differences.

### 3.3 Community Composition

**FIGURE 4:**
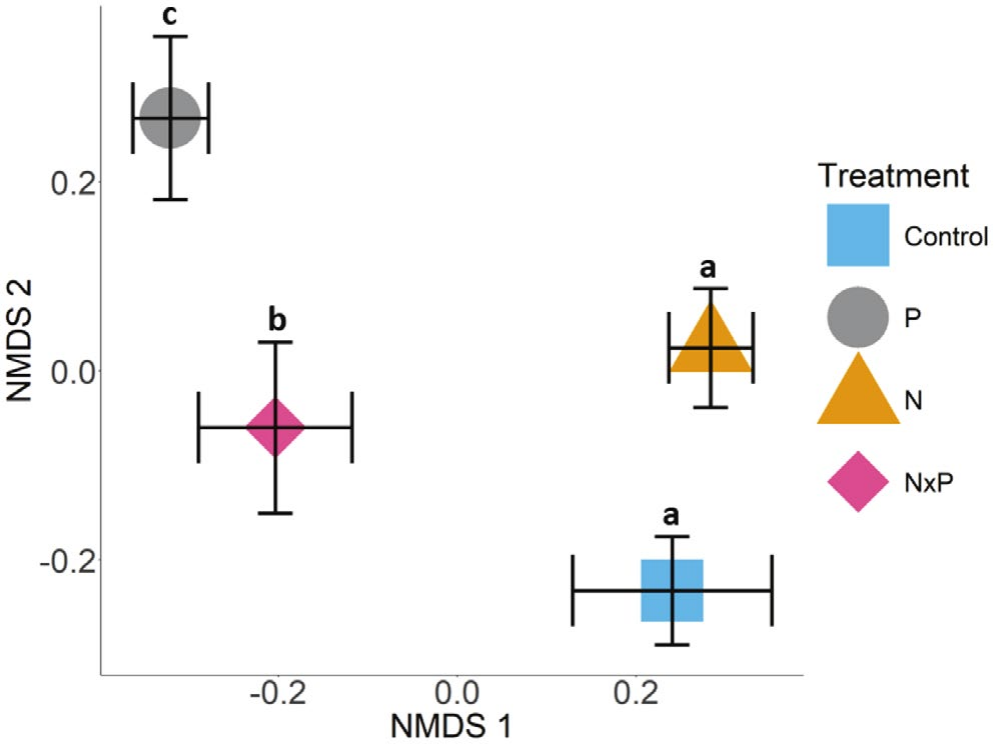
NMDS ordination of treatment‐level community composition. The average NMDS value for each treatment is represented by different colored shapes (control = blue square, nitrogen (N) addition = gold triangle, phosphorus (P) addition = gray circle, and N × P addition = pink diamond). Black bars represent the standard error for NMDS axis 1 and 2. Letters above group centroids represent significant differences.

**FIGURE 5:**
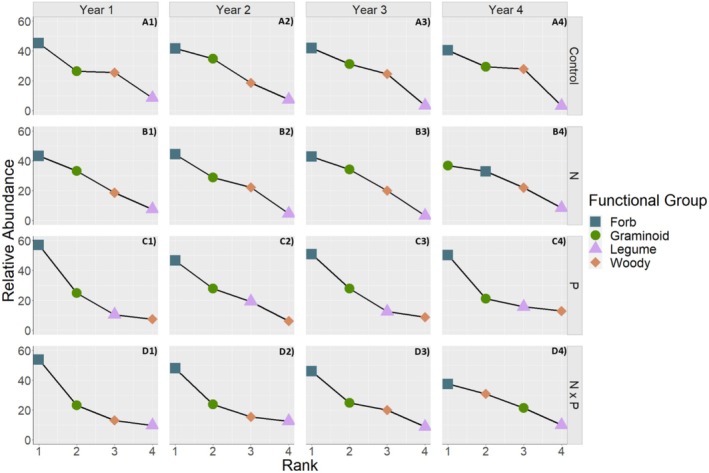
Rank abundance curves through time (years 1–4 from left to right) for control plots (A1–A4), N addition plots (B1–B4), P addition plots (C1–C4), and N × P plots (D1–D5). Each color and shape represent a different functional group (blue square = forb, green circle = graminoid, purple triangle = legume, and orange diamond = woody). Rank 1 represents the highest relative abundance while rank 4 represents the functional group with the lowest relative abundance.

Nutrient addition treatments had a significant effect on understory plant community composition (Figure 4, *F* = 4.9229, *p* = 0.001), accounting for ~25% of the variation in community composition, with no significant interaction between nutrient addition treatments and year. Treatment group centroids were all significantly different from each other, except for control and N addition plots (*p* = 0.17). Finally, there was no significant difference in dispersion around treatment group centroids (*p* = 0.23), indicating that the significant difference in groups' centroids is due to the different nutrient addition treatments and not dispersion. There were six indicator species in control plots (two clonal woody shrubs, two non‐clonal woody shrubs, one clonal forb, and one non‐clonal forb), four indicator species in P plots (one clonal legume, one non‐clonal legume, and two clonal forbs), and nine indicator species in N × P plots (two clonal woody species, one clonal forb, two non‐clonal forbs, three clonal legumes, and one clonal graminoid species) (Appendix S4: Table S3). The ranking of functional groups remained constant through time for control plots (Figure 5A1–A4), with forbs being dominant, followed by graminoids, woody species, and then legumes. Similarly, in phosphorus plots, functional group rankings were constant through time (Figure 5B1–B4) and were dominated by forbs and graminoids, although legumes were ranked third and woody species were ranked last. In both N and N × P addition plots, functional groups were ranked in the same order as control plots until year 4 when there was a reordering of functional groups. In year 4, in N addition plots, graminoids switched rankings with forbs (Figure 5B4), and in N × P addition plots, woody species switched ranking with graminoid species (Figure 5D4).

## 4 Discussion

As hypothesized, N and P additions were each associated with a significant increase in total understory biomass; however, there was no additive effect of N and P together (N × P). With an increase in groundcover biomass, we expected our diversity metrics to also show a response to nutrient additions. Grasslands typically exhibit decreases in biodiversity in response to fertilization, particularly with N additions (Hautier et al. 2009; Muehleisen et al. 2023), a response that has been reported in longleaf pine savannas as well (Kirkman et al. 2016). Frequently, nutrient additions cause an increase in the abundance of more competitive, faster growing, or larger‐stature species, such as bunchgrasses; therefore, richness decreases as smaller forb species are outcompeted for above‐ and belowground resources (Hautier et al. 2009). Alternatively, a meta‐analysis found that the response to fertilization is dependent on how even communities are to begin with; fertilization favors species that are already at a competitive advantage, and more even communities either increase in richness or have no response to fertilization (Hillebrand et al. 2007). This finding for more even communities likely explains why our study found no significant main effect of N, P, or their interaction on richness, diversity, evenness, or dominance compared to control, as our control plots are not highly dominated (Evar ~ 0.45).

While overall biomass increased with each nutrient addition, functional groups showed differential responses. Contrary to our expectations, we found that P does not significantly limit the growth of graminoid or woody species. As graminoid leaf growth is localized in basal meristems, the P requirements for the growth of the remaining part of the leaf are much less than for forbs (Halsted and Lynch 1996), so although the xeric soils of our study site in the Sandhill ecoregion are P depleted, the demand for N over P is greater. In nutrient‐depleted soils, woody species can exhibit tradeoffs between growth and resource storage (Chave et al. 2009), so while woody species did not respond to P additions with increased biomass growth aboveground, there could have been increased growth belowground. Classic co‐limitation, where the addition of each nutrient alone does not significantly increase growth, while adding both does (Liebig 1855; Craine and Jackson 2010), is common across ecosystems globally (Elser et al. 2007). Although N and P were both limiting for most functional groups in this study, there was only evidence for classic co‐limitation in forbs, with an additive increase in biomass with N × P additions, as hypothesized. While the linear models for total, graminoid, legume, and woody biomass did not show a significant interaction effect between N and P, there were observable differences in biomass and significant pairwise differences in biomass between treatments for some functional groups (nonsignificant effect of N × P in the overall model while still having significant pairwise comparisons could potentially be due to the effect being masked by variability within other groups or small sample size). Based on patterns of limitation explained by Craine and Jackson (2010), total biomass exhibited evidence of co‐limitation with substitution, as biomass was more co‐limited by N × P than by N or P alone (i.e., N and P were not different from control and N × P was significantly greater than control and P, but not different from N). Legume biomass, in contrast, exhibited evidence of single‐resource limitation, meaning that there was a just as much of a significant increase in biomass with P compared to control and N × P compared to control. Given that biomass growth in this study is limited by different nutrients for different functional groups, the lack of biodiversity response to nutrient additions means there is likely niche complementarity, where species in the understory can coexist because they are primarily using different resources (MacArthur 1955; Levine and HilleRisLambers 2009). Thus, while we acknowledge that some species may share nutrient requirements and that demographic responses may take longer to detect, the functional divergence in nutrient limitation patterns supports the idea that niche complementarity, rather than strong competitive exclusion, helps maintain coexistence in this diverse understory. In addition to being primarily limited by different resources, species could be using different forms of nutrients in the soil or acquiring resources from different soil depths (McKane et al. 2002; van Ruijven and Berendse 2005). Fire, a common disturbance in longleaf pine savannas, can alter soil structure and nutrient dynamics, potentially influencing how and where different species access resources (Agbeshie et al. 2022). Our study shows that in xeric longleaf pine savannas, high levels of biomass can maintain low levels of competition and high biodiversity in the short term, likely because forb and graminoid species are primarily limited by different soil resources.

Longleaf pine savannas are often thought to have evolved to low nutrient conditions, with dominant bunchgrasses not being limited by nutrients. In fact, another study in longleaf pine savannas found no significant change in productivity and a decrease in graminoid biomass with N addition in xeric sites (Kirkman et al. 2016), such as the one in this study. However, our study finds that overall graminoid biomass was greater with N addition and that understory productivity in general is indeed nutrient limited. This disparity is likely because the previous study averaged productivity responses over a longer time scale (> 10 years) (Kirkman et al. 2016). Systems can respond to a resource press in the short term by increasing in biomass and then in the long term by a change in species composition (Hierarchical Response Framework; Smith et al. 2009). Once composition is altered, productivity may no longer be increased. Although richness did not decrease, the plant community composition did change (i.e., there were the same number of species across treatments, but their identities were different), highlighting that common biodiversity metrics may not fully capture changes in composition that are critical for conservation. This underscores the need for nuanced evaluation of restoration and management success beyond simple diversity counts, especially in nutrient‐poor systems like longleaf pine savannas. Our control, P, and N × P treatment plots were each significantly associated with several indicator species. At our study site, and likely in the Sandhills ecoregion, the presence of clonal and non‐clonal woody shrubs that can acquire resources from a larger area and store more belowground is indicative of resource limitation (Song et al. 2013). Release from nutrient limitation in other grasslands and savannas typically leads to an increase in annual herbaceous species as they rapidly take advantage of the increased resources and outcompete their slow‐growing perennial counterparts (Suding et al. 2005; Xia and Wan 2008; Koerner et al. 2016). However, the majority of the indicator species for P and N × P plots were also clonal perennial species, with only one annual indicator species for any nutrient addition treatment. The lack of annual species post‐fertilization is likely due to the conditions of longleaf pine savannas (frequent fire and sandy, nutrient‐poor soils) that better support perennials in the species pool, as annuals typically do not have the same resilience to fire and require more fertile soils with stable moisture conditions (Grime 1977). It is also possible that nutrient additions can lead to a loss of rare plant species in xeric longleaf pine savannas, as they likely evolved under nutrient‐poor conditions. While composition did change at our site, there were no rare plant species in either the control or treatment communities. However, in systems where rare species are present, the release from nutrient limitation may shift competitive dynamics in ways that reduce the persistence of these rare species, potentially leading to declines in community diversity (Clark and Tilman 2008).

There is extensive literature from grasslands globally showing that increased dominance of C_4_ grasses can competitively exclude other species, decreasing diversity (Grman et al. 2021; Hernández et al. 2022), a phenomenon also observed on mesic soils (Freeman and Trotta 2024) and across multiple soil types of longleaf pine savannas spanning the Sandhill and Coastal Plain ecoregions of North Carolina (Young and Koerner 2022). However, it seems this competitive exclusion is not occurring in xeric longleaf pine savanna sites, like the site in this study. Many studies in longleaf pine savannas show no negative effects of dominant C_4_ grasses on richness (Kirkman et al. 2001; Roth et al. 2008; Myers and Harms 2009), and even suggest the C_4_ bunchgrass, *Aristida stricta*, can have a facilitative effect on other species (Iacona et al. 2012), enabling coexistence. While bunchgrasses can competitively exclude other species as they grow, bunchgrasses are also an essential part of the vegetation‐fire feedback loop that enhances the biodiversity of the understory. In the context of restoration or early successional stages when vegetation structure is still developing, promoting the establishment and increased abundance of C_4_ grasses can help restore this feedback by facilitating fire spread, even on more mesic soils, eventually promoting a diversity of native groundcover in a cyclic process. Frequent fire combined with strategic selection of focal fire‐spreading plant species is therefore essential to a process‐based restoration approach aimed at reestablishing the structure, function, and long‐term sustainability of longleaf pine savannas (Boring et al. 2017; Kirkman and Giencke 2017). It is important to note that nutrient additions during these early stages may assist in establishing fire‐adapted grasses and accelerating restoration trajectories; however, fertilization may have limited benefits for established grass populations, where competitive interactions and community structure are already well established. Fires (both prescribed and unintentional) occur frequently in longleaf pine savannas, leading to persistent soil nutrient deficits (Lavoie et al. 2010). While our results show that N and P additions increased graminoid and forb biomass without reducing overall species richness, the ecological and management implications are complex. For example, many game species rely on the abundance of native forbs and legumes for forage and native bunchgrasses for nesting cover or brood‐rearing habitat (Martin et al. 2012; Palmer et al. 2017), so the increases in biomass of these functional groups with nutrient additions in our study seem beneficial. However, increased N availability also significantly increased woody plant biomass, which is generally undesirable in longleaf pine savannas as it conflicts with management objectives aimed at maintaining open understories. While woody encroachment is often a concern in longleaf pine savanna management, many healthy understories naturally include a low‐statured woody component, typically composed of scrub oaks such as *Quercus laevis* , *Q. marilandica* , or *Q. margarettae* (Peet 2006). However, excessive woody growth, especially when stimulated by nutrient enrichment, can alter fuel structure, reduce herbaceous diversity, and shift the system away from its characteristic open‐canopy state (Gilliam and Platt 1999; Walker and Silletti 2006). Frequent fire is a key mechanism for maintaining low woody abundance in longleaf pine savanna understories (Glitzenstein et al. 2003; Peet 2006), but fertilization that enhances woody growth, even at low levels, can undermine fire effectiveness in controlling woody encroachment. This could promote a shift in competitive dynamics in favor of woody species, with potential long‐term implications for community composition and structure of these savannas. Legumes, which are common in fire‐dependent longleaf pine savannas, can form tripartite, nutrient‐based symbioses with rhizobia and arbuscular mycorrhizal fungi, that enable the legume to incorporate N and P into its tissues (Sprent 2001; Chalk et al. 2006). When engaged in these mutualisms, legumes in longleaf pine savannas could benefit understory productivity by releasing N and P back to the understory through leaf litter and root decomposition, as well as root exudates (Daudin and Sierra 2008; Fustec et al. 2010). Utilizing natural sources of nutrients such as these in restoration could be more cost effective and environmentally friendly alternatives to fertilizers, further emphasizing the importance of plant selection in understory restoration projects. While these findings are likely specific to the soil type in our study, the Sandhills account for some of the largest intact areas of remaining longleaf pine habitat (Oswalt et al. 2012) and are important habitats for restoration and conservation efforts. Our multiyear, chronic nutrient addition field experiment demonstrated that xeric Sandhill longleaf pine savannas can exhibit distinct responses to fertilization compared to other grasslands and savannas globally. Specifically, nutrient additions increased understory productivity without reducing plant biodiversity. However, increased productivity alone does not reliably translate to ecological benefit, and unchanged species richness may mask shifts in composition or losses of nutrient‐sensitive taxa. As such, the ecological implications of nutrient enrichment are likely to be context‐dependent and tied to specific management goals. While we do not advocate for large‐scale fertilization as a management strategy, our findings suggest that low‐level, chronic nutrient inputs (such as those arising from biological nitrogen fixation or atmospheric deposition) may influence community structure in ways that align with some management goals, such as increasing forage biomass or promoting fire spread. Simultaneously, these inputs may pose challenges to other goals, such as limiting woody encroachment or conserving rare, low‐nutrient‐adapted species. Understanding these trade‐offs is important for anticipating how nutrient inputs, whether natural or anthropogenic, may interact with other management goals. Future work should assess whether these patterns persist over longer timescales and vary across different environmental conditions, management practices, and restoration contexts.

## Author Contributions

**Alyssa L. Young:** data curation (lead), formal analysis (lead), visualization (lead), writing – original draft (lead), writing – review and editing (lead). **Kathryn J. Bloodworth:** data curation (equal), visualization (supporting), writing – review and editing (equal). **Page A. Turner:** data curation (supporting), visualization (supporting), writing – review and editing (equal). **Sally E. Koerner:** data curation (supporting), formal analysis (supporting), funding acquisition (lead), visualization (supporting), writing – review and editing (equal).

## Conflicts of Interest

The authors declare no conflicts of interest.

## Supporting information


**Appendix S1:** Methods for drought experiment and non‐significant drought results.
**Appendix S2:** Statistical table showing the main and interacting effects of drought, nutrient addition (NPK), and year on plant biomass (total, forb, graminoid, legume, and woody biomass), based on linear mixed‐effects models.
**Appendix S3:** Statistical table showing the main and interacting effects of drought, nutrient addition (NPK), and year on species richness, Shannon diversity index, Berger–Parker dominance index, and evenness (Evar).
**Appendix S4:** Table of indicator species significantly associated with each treatment group.

## Data Availability

Data and code used for this study are available via GitHub (https://github.com/alyoung6720/NutientLimitationLongleafPine2024).
