# Supplementary material for: Evidence of Nitrogen and Phosphorus Limitation in Longleaf Pine Savanna Understories
Source: Ecol Evol. 2025 Sep 21;15(9):e71836. doi: 10.1002/ece3.71836 (PMC12450610; doi:10.1002/ece3.71836)
Supplement: Supplementary file 1 — Appendix S1: Methods for drought experiment and non‐significant drought results. Appendix S2: Statistical table showing the main and interacting effects of drought, nutrient addition (NPK), and year on plant biomass (total, forb, graminoid, legume, and woody biomass), based on linear mixed‐effects models. Appendix S3: Statistical table showing the main and interacting effects of drought, nutrient addition (NPK), and year on species richness, Shannon diversity index, Berger–Parker dominance index, and evenness (Evar). Appendix S4: Table of indicator species significantly associated with each treatment group. [file ECE3-15-e71836-s001.docx]

**Supplementary Material for Young et al.**

**Appendix S1**

Drought Methods

Drought shelters were constructed and erected in a control and an NPK plot within each of the 3 blocks (n=6). For each 5x5 m plot, metal t-posts were pounded into the ground in the middle and at ~1m outside of each corner of the plot so the drought shelter would cover a 6x6 m area above the plot. Metal poles were arranged in a 6x6 m square and attached to the posts above the vegetation layer. Clear plastic roofing was then attached to the metal square so that ~29% of rainfall would be reduced over the whole plot. The percent reduction in rainfall was intended to mimic an extreme drought which was determined by finding the 1^st^ percentile of a long-term precipitation record over the last 100 years. Gutters with black tubing on each end were then attached to the metal poles so that water falling down the plastic roofing would be collected in the gutter and diverted away from the field site in plastic tubing. The drought treatment lasted for 4 years of the experiment (2020-2023) and was erected and deconstructed as follows: erected in June and deconstructed in October of year 1, erected in May and deconstructed in September of year 2, erected in April and deconstructed in September of year 3, and erected in April and deconstructed in July of year 4. In each drought × NPK plot, nitrogen (N), phosphorus (P), and potassium (K) each at a rate of 10g m^-1^yr^-1^ were administered yearly for 4 years (2020-2023) during the early growing season. Plant community composition and aboveground biomass in drought plots were sampled the same way as nutrient addition plots, as described in the methods.

Drought Results

Drought had no significant effects on any of our biodiversity metrics (richness, diversity, dominance, evenness, wiregrass cover) or total, forb, graminoid biomass, or woody biomass. Interestingly, drought and year interacted with legume biomass, with drought plots having on average 169% less legume biomass than control in year 2.

**Appendix S2**


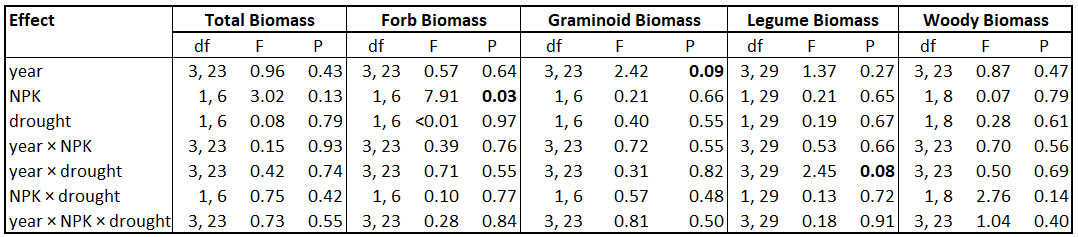
**Table S1.** Main and interacting effects of drought, NPK, and year (fixed effects) from linear mixed models on total biomass (sum of forb, graminoid, legume, and woody biomass), forb biomass, and graminoid biomass. Block and plot were included as random effects. Significant p-values (P < 0.10) are bolded.

**Appendix S3**

**Table S2.** Main and interacting effects of drought, NPK, and year (fixed effects) from linear mixed models on species richness, Shannon diversity index, Berger-Parker dominance index, and evenness (Evar). Block and plot were included as random effects. Significant p-values (P < 0.10) are bolded.

**Appendix S4**

Table S3. Indicator species that are significantly associated with each treatment group. Species are categorized based on if they are clonal (yes or no), functional group (graminoid, forb, legume, or woody), lifespan (annual or perennial), and presence in control, N, P, and N×P treatment groups (denoted by ‘X’).
